# Clinical characteristics and risk factors associated with *Pneumocystis jirovecii* infection in patients with solid tumors: study of thirteen-year medical records of a large cancer center

**DOI:** 10.1186/s12885-021-08727-2

**Published:** 2021-09-03

**Authors:** Koichi Takeda, Sohei Harada, Brian Hayama, Kosuke Hoashi, Taisuke Enokida, Toshiharu Sasaki, Koh Okamoto, Kenji Nakano, Daisuke Ohkushi

**Affiliations:** 1 Department of Infectious Diseases, Cancer Institute Hospital, Japanese Foundation for Cancer Research, 3-8-31 Ariake, Koto-ku, Tokyo, 135-8550 Japan; 2 Department of Infection Control and Prevention, The University of Tokyo Hospital, 7-3-1 Hongo, Bunkyo-ku, Tokyo, 113-8655 Japan; 3 Department of Infectious Diseases, Iizuka Hospital, 3-83 Yoshio-machi, Iizuka, Fukuoka, 820-8505 Japan; 4 Department of Infectious Diseases, Fujita Health University School of Medicine, 1-98 Dengakugakubo, Kutsukake-cho, Toyoake, Aichi 470-1192 Japan; 5 Department of Infectious Diseases, The University of Tokyo Hospital, 7-3-1 Hongo, Bunkyo-ku, Tokyo, 113-8655 Japan; 6 Department of Medical Oncology, Cancer Institute Hospital, Japanese Foundation for Cancer Research, 3-8-31 Ariake, Koto-ku, Tokyo, 135-8550 Japan

**Keywords:** Beta-D-glucans, Corticosteroids, Invasive fungal infections, Mycoses, Solid tumors, *Pneumocystis jirovecii*

## Abstract

**Background:**

*Pneumocystis jirovecii* pneumonia (PCP)-related risk factors among patients with solid tumors are not completely defined. Thus, we aimed to characterize PCP cases with underlying solid tumors, to highlight the factors contributing to its development besides the prolonged use of moderate-to-high dose corticosteroids.

**Methods:**

We retrospectively reviewed the medical records of patients with solid tumors diagnosed with PCP between 2006 and 2018 at a cancer center in Tokyo, Japan. Demographic and clinical data were collected, which included malignancy types, total lymphocyte count, coexisting pulmonary disease, chemotherapy, radiation therapy, corticosteroid use, and PCP-attributable mortality.

**Results:**

Twenty cases of PCP with solid tumors were documented in 151,718 patients and 788,914 patient-years. Lung cancer (*n* = 6, 30%) was the most common underlying tumor, followed by breast cancer (*n* = 3, 15%). Only six (30%) patients were taking a dosage of ≥20 mg prednisone equivalents daily for ≥4 weeks from the onset of PCP. Among the remaining 14 patients, seven (50%) had coexisting pulmonary diseases, 10 (71%) had received chemotherapy within 90 days prior to PCP diagnosis, seven (50%) had undergone chest radiation therapy before PCP diagnosis, seven (50%) had received only intermittent corticosteroids, and one (7%) received no corticosteroids. Mortality attributable to PCP was 40%.

**Conclusions:**

More than half of the patients were not taking a dosage of ≥20 mg prednisone equivalents daily for ≥4 weeks. Multiple other factors (e.g., lymphocytopenia, radiation to chest) may have potentially contributed to PCP in patients with solid tumors in a composite manner. We need to establish a method for estimating the likelihood of PCP taking multiple factors into account in this patient population.

## Background

*Pneumocystis jirovecii* pneumonia (PCP) is common among patients with solid tumors. The PCP incidence in this population is approximately half of that in patients with hematological malignancies [1, 2]. However, the risk factors for PCP in patients with solid tumors have not been well defined.

Current international guidelines recommend primary PCP prophylaxis for patients with solid tumors, when the prolonged use of a moderate-to-high corticosteroid dose (i.e., with ≥20 mg prednisone equivalents daily for ≥4 weeks) or when temozolomide with radiation therapy is adopted [3–5]. Nevertheless, PCP occurs without these well-known risk factors in patients treated with intensive cancer chemotherapy. The chemotherapy regimens associated with PCP occurrence include adriamycin/cyclophosphamide-containing regimen and weekly paclitaxel and trastuzumab regimen for breast cancer [6, 7], everolimus-containing regimen for breast cancer and renal cancer [8–10], FOLFIRINOX (oxaliplatin, irinotecan, leucovorin, and 5-fluorouracil) chemotherapy for pancreatic cancer [11], and gemcitabine chemotherapy for breast and pancreatic cancers [12]. Other factors such as radiation therapy [13, 14], intermittent courses of corticosteroids during chemotherapy (as antiemetics and/or premedication for hypersensitivity reactions) [15, 16], lymphopenia [13], coexisting pulmonary disease, and immunosuppression associated with coexisting disease (underlying disease itself or immunosuppressive therapy against the disease) may also influence PCP risk.

We aimed to characterize PCP cases with underlying solid tumors by performing a retrospective descriptive study at a cancer center, with special emphasis on cases without prolonged use of a moderate-to-high corticosteroid dose, to determine the additional PCP risk factors in these cases.

## Methods

### Study design and settings

This was a retrospective observational study performed at a 686-bed tertiary care cancer center in Tokyo, Japan, between January 2006 and December 2018. The center has approximately 13,000 annual admissions of patients with solid tumors.

### Study population and inclusion criteria

We reviewed the medical records of all adult patients (≥ 18 years) who tested positive for *Pneumocystis*-specific qualitative polymerase chain reaction (PCR) performed on respiratory specimens (e.g., sputum, induced sputum, and bronchoalveolar lavage fluid) during the study period. This analysis was performed commercially at SRL Inc. (Shinjuku, Japan) by using the previously described methods as part of the diagnostic procedure in daily practice [17].

Among these patients, we selected patients with (1) a diagnosis of solid tumors, (2) pulmonary radiographic findings compatible with PCP, and a (3) plasma beta-D-glucan (BDG) (Wako Pure Chemical Industries, Tokyo, Japan) value ≥11 pg/mL within the 14-day period, starting 7 days prior to the day of the initial radiographic study showing pulmonary infiltrates. Plasma BDG was used to exclude cases with probable colonization of *P. jirovecii* in the respiratory tract without causing pneumonia. Patients were excluded if they had (1) concomitant hematological malignancies, (2) known Human Immunodeficiency Virus (HIV) infection, (3) received unapproved cancer drugs during PCP diagnosis, or (4) have been participating in blinded clinical trials and were unaware of the drugs they were taking. Patients were also excluded if they had conditions that are possibly associated with elevated plasma BDG levels unrelated to PCP, including (5) receiving blood components or plasma protein products (e.g., intravenous immunoglobulins, albumin), hemodialysis, or surgery within 7 days prior to plasma BDG sampling [18, 19] or (6) having other concomitant fungal infections, as defined by the 2008 consensus definitions of the European Organization for the Research and Treatment of Cancer/Mycoses Study Group, within a 28-day period starting 14 days prior to the day of the plasma BDG sampling [20].

### Data collection

The number of patients with solid tumors in the outpatient clinic of the hospital during the study period was extracted from the hospital database to estimate the incidence of PCP in this patient population.

The demographic and clinical data of patients diagnosed with PCP with underlying solid tumors were collected via a chart review. The data included age, sex, malignancy types, PCP prophylaxis within 90 days prior to the diagnosis of PCP, total lymphocyte count, serum lactate dehydrogenase (LDH), plasma BDG, radiological findings, coexisting pulmonary disease, past chemotherapies, time interval from the last chemotherapy to the diagnosis of PCP, radiation of the chest (field and cumulative dose), time interval from the last radiation therapy to the diagnosis of PCP, corticosteroid use (duration and cumulative dose) within 90 days prior to PCP diagnosis, reason for corticosteroid use, pattern of corticosteroid administration (daily or intermittently), use of immunosuppressive agent within 90 days prior to PCP diagnosis, severity of PCP, treatment regimen for PCP (choice of drugs and duration), mortality attributable to PCP, and 30-day overall mortality.

The day of PCP diagnosis was defined as the first day when relevant pulmonary infiltrates were documented by chest imaging. Data on total lymphocyte count and serum LDH and plasma BDG levels were collected on the day of (or the closest day within 7 days prior to) PCP diagnosis. Board-certified radiologists evaluated the chest images. Coexisting pulmonary diseases were defined as interstitial pneumonia, chronic obstructive pulmonary disease, bronchiectasis, healed pulmonary tuberculosis, primary/metastatic lung tumors, and radiation pneumonitis. The corticosteroid dosage was expressed in prednisone equivalents (we calculated dexamethasone or betamethasone dosage of 1 mg as equivalent to prednisone dosage of 7.5 mg) [21]. We included cyclophosphamide, azathioprine, mycophenolate mofetil, methotrexate, cyclosporine, and tacrolimus as immunosuppressive agents. However, if cyclophosphamide and methotrexate were used as part of a chemotherapy regimen, we did not consider them as immunosuppressive agents. Biological agents and small molecule kinase inhibitors used in the management of patients with concomitant autoimmune disorders were also considered immunosuppressive agents. PCP severity was considered “moderate to severe” in patients with arterial–alveolar difference > 35 mmHg, arterial oxygen pressure < 70 mmHg, or ambient air O_2_ saturation < 92%. PCP severity was considered “mild” in patients not meeting these criteria. Mortality was attributed to PCP if death was due to progressive respiratory failure in a PCP patient.

## Results

**Fig. 1:**
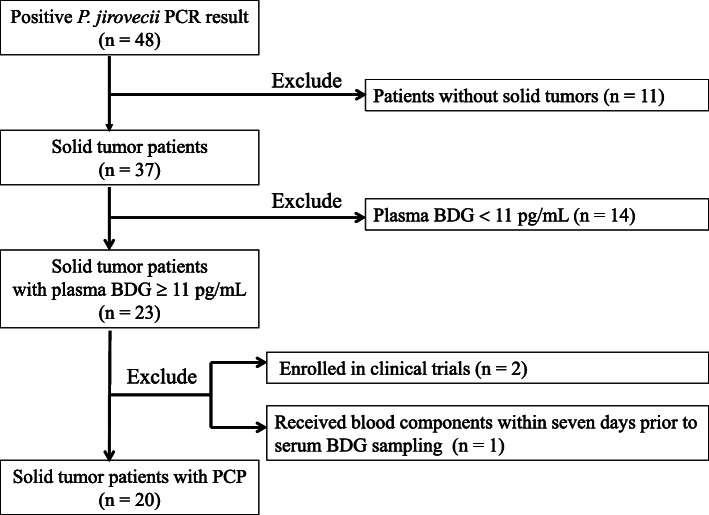
Flowchart showing the inclusion and exclusion criteria applied in the selection of the study population. *P. jirovecii, Pneumocystis jirovecii*; PCR, polymerase chain reaction; BDG, beta-D-glucan; PCP, *Pneumocystis jirovecii* pneumonia

During the 13-year study period, the respiratory specimens of 48 patients tested positive for *Pneumocystis*-specific qualitative PCR. Among these patients, 23 patients fulfilled the inclusion criteria, but 3 of them were later excluded because they also met the exclusion criteria. Thus, a total of 20 patients were analyzed in our study (Fig. 1). During the study period, the hospital cared 151,718 patients and 788,914 patient-years with solid tumors. The calculated incidence of PCP among patients with solid tumors at the hospital was 13.2 per 100,000 patient and 2.54 per 100,000 patient-years.

### Baseline and radiologic characteristics of the PCP patients

**Fig. 2:**
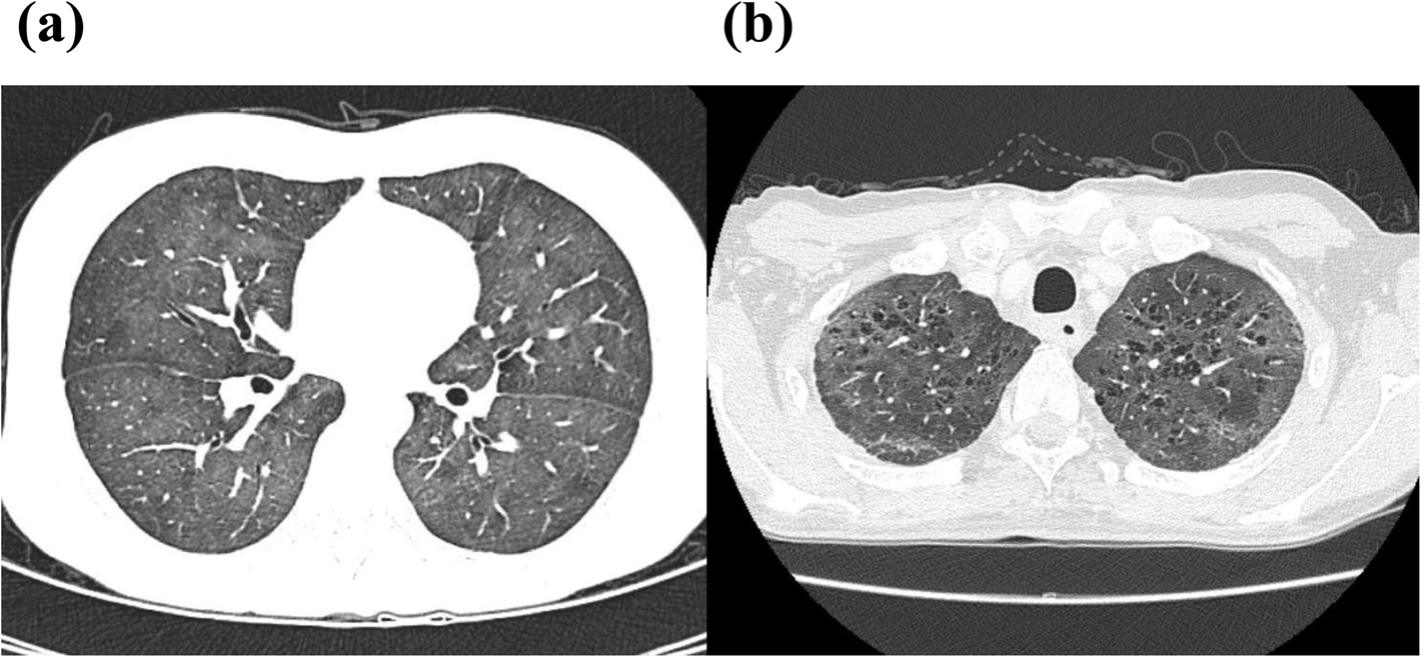
Chest CT findings in patients with ground glass opacities. Patient No. 13 (**a**) and Patient No. 14 (**b**) shown in Table 3

The clinical characteristics of the 20 PCP patients are separately shown according to the prolonged use of a moderate-to-high corticosteroid dose (i.e., with or without a dosage ≥20 mg prednisone equivalents daily for ≥4 weeks) (Table 1). Fourteen (70%) were patients without prolonged use of a moderate-to-high corticosteroid dose. Among the 20 patients, none had received primary prophylaxis for PCP. According to cancer type, lung cancer (*n* = 6, 30%) was the most common, followed by breast cancer (*n* = 3, 15%). The remaining 11 patients had other diverse cancer types. Eighteen patients (90%) developed PCP while their lymphocyte counts were below 1000 cells/mm^3^. Ground glass opacities, which are typical findings of PCP, were observed in 18 patients (90%) on chest CT (Fig. 2).

**Table 1:** Clinical characteristics of the patients with *P. jirovecii* pneumonia

	Patients with prolonged use of a moderate-to-high corticosteroid dose (*n* = 6)	Patients without prolonged use of a moderate-to-high corticosteroid dose (*n* = 14)	Total (*n* = 20)
Age (years), median (range)	56 (46–76)	67 (40–90)	66 (40–90)
Cancer type, n (%)			
Lung cancer	1 (17)	5 (36)	6 (30)
Breast cancer	1 (17)	2 (14)	3 (15)
Esophageal cancer	0 (0)	2 (14)	2 (10)
Pancreatic cancer	1 (17)	1 (7)	2 (10)
Others^a^	3 (50)	4 (29)	7 (35)
ALC, n (%)			
< 500	3 (50)	9 (64)	12 (60)
500–999	2 (33)	4 (29)	6 (30)
≥ 1000	1 (17)	1 (7)	2 (10)
LDH (Units/L)			
< 250	1 (17)	2 (14)	3 (15)
250–500	4 (67)	9 (64)	13 (65)
> 500	1 (17)	3 (21)	4 (20)
BDG, median (range)	85.8 (19.8–522.1)	37.8 (16− > 600)	45 (16− > 600)
Main Radiologic findings, n (%)			
Ground glass opacities	5 (83)	13 (93)	18 (90)
Other^b^	1 (17)	1 (7)	2 (10)

*ALC* Absolute lymphocyte count, *LDH* Lactate dehydrogenase, *BDG* Beta-D glucan, *PSL* Prednisolone

^a^ Rectal cancer, thyroid cancer, and inflammatory myofibroblastic tumor with single case each in patients with PSL ≥ 20 mg daily ≥4 weeks and ovarian cancer, renal cancer, laryngeal cancer, and hypopharyngeal cancer with a single case each in patients without PSL ≥ 20 mg daily ≥4 weeks

^b^ Examples of other findings were patchy, nodular, and focal consolidation

### Treatment outcomes of PCP

According to the definition, 9 patients (45%) had mild PCP, and 11 patients (55%) had moderate to severe PCP at the time of initial presentation (Table 2**)**. All patients received adequate initial therapy; 18 patients received trimethoprim and sulfamethoxazole (TMP-SMX), and 2 patients with mild PCP received atovaquone as a first-line therapy. Nine patients who were initially treated with TMP-SMX were switched to second-line medication (intravenous pentamidine in 4 cases and atovaquone in 5 cases) owing to the adverse effects of TMP-SMX therapy. The duration of PCP treatment was 2 to 3 weeks in all cases. Fifteen patients (75%) received adjunctive corticosteroids (either newly initiated or increased from baseline dosage) during PCP treatment. Mortality attributable to PCP and 30-day overall mortality were 8/20 (40%) and 6/20 (30%), respectively.

**Table 2:** Treatment outcomes for PCP

	Patients with prolonged use of a moderate-to-high corticosteroid dose (*n* = 6)	Patients without prolonged use of a moderate-to-high corticosteroid dose (*n* = 14)	Total (*n* = 20)
Treatment regimen, no. (%)			
TMP-SMX only	2 (33)	7 (50)	9 (45)
TMP-SMX prior to intravenous pentamidine	3 (50)	1 (7)	4 (20)
TMP-SMX prior to atovaquone	1 (17)	4 (29)	5 (25)
Atovaquone only	0	2 (14)	2 (10)
Adjunctive corticosteroids, no. (%)			
Not used	0	2 (14)	2 (10)
Newly initiated	0	6 (43)	6 (30)
Unchanged from baseline dosage	2 (33)	0	2 (10)
Increased from baseline dosage	3 (50)	6 (43)	9 (45)
Decreased from baseline dosage	1 (17)	0	1 (5)
Severity of PCP, no. (%)			
Mild	2 (33)	7 (50)	9 (45)
Moderate to severe	4 (67)	7 (50)	11 (55)
Mortality attributed to PCP, no. (%)	5 (83)	3 (21)	8 (40)
Thirty-day mortality, no. (%)	3 (50)	3 (21)	6 (30)

*TMP-SMX* Trimethoprim-sulfamethoxazole, *PCP Pneumocystis jirovecii* pneumonia

### Analysis of 14 patients who developed PCP without prolonged use of a moderate-to-high corticosteroid dose

The clinical characteristics of the 14 patients who developed PCP without prolonged use of a moderate-to-high corticosteroid dose (i.e., without ≥20 mg prednisone equivalents daily for ≥4 weeks) are shown in Table 3. At the time of PCP diagnosis, 7 patients (50%) had coexisting pulmonary diseases. Chemotherapy regimens were diverse, and no specific regimen was dominant. The number of patients receiving their last chemotherapy between 90 and 30 days prior to the diagnosis of PCP was 10 (71%) and 7 (50%), respectively. During the 90 days prior to PCP diagnosis, 6 patients (43%) had received daily corticosteroids without fulfilling the criteria of “prolonged use of a moderate-to-high corticosteroid dose” in terms of daily dosage and/or duration, 7 patients (50%) had received only intermittent corticosteroids, and 1 patient (7%) had received no corticosteroids. During the 90 days prior to PCP diagnosis, only 4 patients (29%) had received a dosage of ≥700 mg prednisone equivalents as a cumulative dose. Only 1 patient (7%) (case 11) had received immunosuppressive agents 90 days prior to PCP diagnosis. This patient had been receiving a methotrexate dosage of 10 mg weekly for coexisting rheumatoid arthritis. Seven patients (50%) had received radiation therapy of the chest before PCP diagnosis, and 3 patients (21%) received therapy within 10 days.

**Table 3:** Clinical characteristics of the 14 patients who developed PCP without prolonged moderate-to-high corticosteroid dose usage

No	Age (years)	Sex	Cancer type	Coexisting pulmonary diseases	CTx before PCP diagnosis	Days from last CTx to PCP diagnosis	Types/cumulative dose^a^/total days of corticosteroid administration within 90 days prior to PCP diagnosis	Reason for corticosteroid use	Field/cumulative dose of radiation therapy	Days from last radiation therapy to PCP diagnosis
1	71	M	Lung cancer	Lung cancer	Gefitinib Erlotinib CDDP/PEM/Bev Gefitinib	0	Daily/630 mg/42 days	Palliative therapy	None	NA
2	90	M	Renal cancer	IP	None	NA	Daily/960 mg/68 days	Spinal cord compression	Thoracic spine/55 Gy	61
3	71	M	Lung cancer	Lung cancer, COPD, radiation pneumonitis	CDDP/VP-16	156	Daily/560 mg/28 days	Radiation pneumonitis	Lung/39 Gy	102
4	64	M	Esophageal cancer	None	CDDP/5-FU	58	Daily/675 mg/38 days Intermittent/223 mg/4 days	Severe drug allergy Antiemetics/premedication for CTx	Esophagus/60 Gy	54
5	70	M	Pancreatic cancer	None	GEM/nabPTX	13	Daily/630 mg/42 days Intermittent/248 mg/5 days	Palliative therapy Antiemetics/premedication for CTx	None	NA
6	64	M	Lung cancer	Lung cancer, radiation pneumonitis	CDDP/S-1	107	Daily/405 mg/20 days	Radiation pneumonitis	Lung/60 Gy	94
7	59	F	Ovarian cancer	None	CBDCA/PTX DOC	33	Intermittent/120 mg/2 days	Antiemetics/premedication for CTx	None	NA
8	60	M	Esophageal cancer	None	5-FU	11	Intermittent/446 mg/8 days	Antiemetics/premedication for CTx	Esophagus/58 Gy	0
9	68	F	Breast cancer	None	ADR/CY	15	Intermittent/387 mg/12 days	Antiemetics/premedication for CTx	None	NA
10	67	M	Lung cancer	Lung cancer	CBDCA/PTX	98	None	NA	Lung/66 Gy	9
11	67	M	Laryngeal cancer	Metastatic lung cancer	PTX/Cet	14	Intermittent/495 mg/10 days	Antiemetics/premedication for CTx	None	NA
12	72	M	Lung cancer	Lung cancer	CBDCA/PTX	64	Intermittent/248 mg/2 days	Antiemetics/premedication for CTx	Lung/52 Gy	3
13	40	F	Breast cancer	None	CY/EPI/5-FU DOC/HER	12	Intermittent/932 mg/14 days	Antiemetics/premedication for CTx	None	NA
14	58	M	Hypopharyngeal cancer	None	CDDP PTX/Cet	5	Intermittent/396 mg/8 days	Antiemetics/premedication for CTx	None	NA

*PCP Pneumocystis jirovecii* pneumonia, *CTx* Chemotherapy, *M* Male, *F* Female, *IP* Interstitial pneumonia, *COPD* Chronic obstructive pulmonary disease, *CDDP* Cisplatin, *PEM* Pemetrexed, *Bev* Bevacizumab, *VP-16* Etoposide, *5-FU* 5-fluorouracil, *GEM* Gemcitabine, *nabPTX* Nab-paclitaxel, *S-1* TS-1, *CBDCA* Carboplatin, *PTX* Paclitaxel, *DOC* Docetaxel, *ADR* Adriamycin, *CY* Cyclophosphamide, *Cet* Cetuximab, *EPI* Epirubicin, *HER* Trastuzumab, *Gy* Gray, *NA* Not available

^a^The dosage of cumulative corticosteroids was expressed in prednisone equivalents

## Discussion

We characterized cases of PCP with underlying solid tumors. Twenty patients developed PCP under no primary prophylaxis. All patients had received adequate initial therapy, and mortality attributable to PCP and 30-day overall mortality were 40 and 30% respectively.

During the 13-year study period, 14 patients developed PCP without prolonged use of a moderate-to-high corticosteroid dose. The mortality from PCP in patients without HIV infection is generally higher than that in HIV-infected patients (~ 35–50%) [22–24]. The prognostic benefit of adjunctive use of corticosteroid, which is well established in PCP in patients with HIV, is uncertain in this patient population [25]. In the current study, the mortality attributable to PCP was 40%, which was similar to previous reports. By taking the high mortality into account, we sought to identify the group of patients with nonnegligible PCP risk among noncandidates for primary prophylaxis under the current guidelines. Thus, we described in detail the background of 14 patients who met these criteria.

In the 14 patients, lung cancer (*n* = 5, 36%) was the most common, followed by breast (*n* = 2, 14%) and esophageal (*n* = 2, 14%) cancers. In a study at a cancer center, primary and metastatic brain tumors were the most common among patients with PCP in the presence of solid tumors [26], followed by lung and breast cancers; this is similar to the findings of the present study. Here, no patient had brain tumors diagnosed with PCP because of the lack of a neurosurgery department at the hospital. There was no predominance of any specific chemotherapy, but many patients (*n* = 10, 71%) received chemotherapy within 90 days. A previous study of 80 PCP episodes in cancer patients revealed that chemotherapy or immunosuppressive drugs had been administered in 50 episodes (63%) within 1 month prior to PCP diagnosis [27]. The recent administration of cancer chemotherapy may be a risk factor for PCP development.

In this study, 7 (50%) of the 14 patients had received chest radiation therapy. In particular, case 10 (lung cancer) received chest radiation therapy (66 Gy totally) within 9 days prior to PCP diagnosis and had no history of cancer chemotherapy or corticosteroid use within 90 days. Therefore, it is presumed that radiation therapy contributed significantly to PCP onset in this patient. In a previous study of 26 patients who developed PCP with underlying solid tumors and lymphomas, 22 patients (85%) had a history of irradiation [13]. Thoracic duct irradiation may contribute to lymphopenia, with radiation therapy at the mediastinal area considered a PCP-predisposing factor [13]. A study of PCP patients with lung cancer also concluded that concurrent chemoradiotherapy was a risk factor for PCP development [14]. Therefore, it may be necessary to consider radiation therapy (particularly for the thorax/lung/mediastinum) as a PCP risk factor.

PCP occurred not only in patients receiving daily corticosteroids but also in seven patients (50%) receiving only intermittent courses of corticosteroids as an antiemetic and/or premedication for hypersensitivity reactions during chemotherapy. In a previous study of 128 HIV-uninfected PCP patients, 50 patients (43%) received intermittent corticosteroids only [16]. It is essential to recognize that PCP can develop in such treatment backgrounds. The risk of infectious disease increased when the cumulative dose of daily corticosteroids exceeded 700 mg (prednisone equivalent) [28]. However, it is unknown whether this threshold can be applied to the cumulative dose of intermittent corticosteroid administration. In fact, among the 7 patients who developed PCP on intermittent corticosteroids, only 1 (case 13) had exceeded a cumulative dose of 700 mg (prednisone equivalent) within 90 days prior to PCP diagnosis. Future studies are required to evaluate the quantitative effect of intermittent corticosteroids on PCP development. Among the 14 patients without prolonged use of a moderate-to-high corticosteroid dose, all but 1 patient (93%) had a lymphocyte count of less than 1000 cells/mm^3^, while 9 patients (64%) had fewer than 500 cells/mm^3^. In a previously mentioned study of 26 patients who developed PCP with underlying solid tumors and lymphomas, 18 patients (69%) had a lymphocyte count of less than 1000 cells/mm^3^, while 9 patients (35%) had fewer than 500 cells/mm^3^ [13]. These data suggest that lymphopenia may be a PCP risk factor. Among the 14 patients without prolonged use of a moderate-to-high corticosteroid dose, 7 patients (*n* = 7, 50%) had coexisting pulmonary diseases. A study of PCP in patients with rheumatoid arthritis who were receiving infliximab therapy, revealed that the presence of coexisting pulmonary disease was one of the PCP risk factors [29]. The spectrum of coexisting pulmonary diseases that contributes to PCP development and their effects remains to be elucidated.

There are several limitations to this study. First, the study included only patients tested with *Pneumocystis*-specific PCR. Owing to the nature of the retrospective study, all suspected PCP patients may not have undergone PCR testing, thus possibly leading to the inclusion of fewer PCP patients than the actual number of cases. However, the incidence rate of PCP among patients with solid tumors here was similar to that among lung or breast cancer patients in a previous report [1]. Therefore, we believe that there is no major undercounting of PCP patients in our cancer center. Second, our study relied heavily on conventional PCR (c-PCR) and plasma BDG for PCP diagnosis because of the lack of commercially available real-time PCR in Japan. Although c-PCR has low specificity in diagnosing PCP because it cannot distinguish colonization from infection; the sensitivity was reported as high as 90% (equivalent to/higher than that of qPCR) [30]. Therefore, we postulated that c-PCR would be suitable for screening patients. Furthermore, given that the specificity of plasma BDG ≥ 11 pg/mL for diagnosing PCP was reported to be very high (98–100%) in selected situations [31, 32] and that this study carefully excluded the known factors that increase plasma BDG levels other than PCP, we assume that the PCP cases in the present study reflect actual cases. Lastly, the lack of controls prevented the statistical assessment of individual factors that may have contributed to PCP development. In addition, since this is a single-center study with a limited number of cases, further research is needed to determine whether the observed results are applicable in different settings.

## Conclusions

Most patients with solid tumors who developed PCP were not taking a moderate-to-high corticosteroid dose for prophylaxis. Multiple factors that are not currently recognized as stand-alone risk factors probably contribute to PCP development in a composite manner are types of solid tumors, recent administration of cancer chemotherapy, chest radiation therapy, low-dose or intermittent corticosteroids and other immunosuppressive drugs, lymphocyte count, and coexisting pulmonary diseases. As stated in latest guidelines, universal prophylaxis of PCP in these patients is not recommended, given the low incidence [5]. Nevertheless, we need to establish a method for estimating the likelihood of PCP taking multiple factors into account in patients with solid tumors with newly appearing pulmonary infiltrates.

## Data Availability

All supporting information can be found in the tables in the article.

## Abbreviations

PCP: *Pneumocystis jirovecii* pneumonia
FOLFIRINOX: Oxaliplatin, Irinotecan, Leucovorin, and 5-Fluorouracil
PCR: Polymerase Chain Reaction
BDG: Beta-D-Glucan
HIV: Human Immunodeficiency Virus
LDH: Lactate Dehydrogenase
TMP-SMX: Trimethoprim and Sulfamethoxazole
c-PCR: Conventional Polymerase Chain Reaction
qPCR: Quantitative Polymerase Chain Reaction
